# The Molecular Impacts of Retrotransposons in Development and Diseases

**DOI:** 10.3390/ijms242216418

**Published:** 2023-11-16

**Authors:** Phoebe Lut Fei Tam, Danny Leung

**Affiliations:** 1 Division of Life Science, The Hong Kong University of Science and Technology, Clear Water Bay, Hong Kong SAR, China; lftamaa@connect.ust.hk; 2 Center for Epigenomics Research, The Hong Kong University of Science and Technology, Clear Water Bay, Hong Kong SAR, China

**Keywords:** retrotransposons, epigenomics, mammalian development, diseases

## Abstract

Retrotransposons are invasive genetic elements that constitute substantial portions of mammalian genomes. They have the potential to influence nearby gene expression through their *cis*-regulatory sequences, reverse transcription machinery, and the ability to mold higher-order chromatin structures. Due to their multifaceted functions, it is crucial for host fitness to maintain strict regulation of these parasitic sequences to ensure proper growth and development. This review explores how subsets of retrotransposons have undergone evolutionary exaptation to enhance the complexity of mammalian genomes. It also highlights the significance of regulating these elements, drawing on recent studies conducted in human and murine systems.

## 1. Retrotransposons

Transposable elements (TEs) make up almost half of the genomes of both mice and humans [1]. These genetic sequences proliferate within host cells through distinct replication mechanisms. Their significance in influencing gene regulation was initially revealed by the pioneering work of Barbara McClintock in the 1950s [2]. However, despite their substantial presence, these genomic sequences were previously regarded as ‘junk’ DNA and largely overlooked in genetics research. With the advent of modern massive parallel sequencing technologies, mounting evidence has underscored their crucial roles in gene regulation.

Retrotransposons are defined as class I TEs, which replicate by copying and pasting themselves throughout the genome in a process called retrotransposition. Akin to retroviruses that have RNA genomes, retrotransposons reverse transcribe their RNA transcripts into double-stranded DNA (dsDNA), which can integrate into different loci of the host genome. Based on the presence of long terminal repeats (LTRs), retrotransposons are divided into LTR retrotransposons and non-LTR retrotransposons. Endogenous retroviruses (ERVs), which comprise 8% and 10% of the human and mouse genome, respectively, are classified as LTR retrotransposons and arise from ancient infections of retroviruses. After integration, the elements, termed proviruses, are identical to their exogenous counterparts, possessing two LTRs flanking the viral *pro*, *gag*, *pol*, and *env* genes. Such proviruses are defined as full-length or complete ERVs and can retrotranspose throughout the genome autonomously. Numerous studies have delineated the mechanism of ERV transcription and reverse transcription [3,4,5,6]; however, the process of second-strand DNA synthesis before integration has not been fully elucidated. A recent study in *Drosophila melanogaster* revealed that ERVs can utilize alternative end joining (alt-EJ), a DNA double-strand break (DSB) repair pathway, to synthesize the second strand through circularization [7]. Non-LTR retrotransposons include long interspersed nuclear elements (LINEs) and short interspersed nuclear elements (SINEs). LINE-1 (L1) retrotransposons are the most active and abundant TEs [8], accounting for approximately 17% of the human genome [9]. These elements have open reading frames (ORF1, ORF2, and the primate specific antisense ORF0), which confer their ability to mobilize through target-primed reverse transcription (TPRT) [10,11,12]. SINEs, on the other hand, constitute around 13% of the human genome [8] and are non-autonomous TEs that lack protein-coding genes [13]. They depend on the transposition machinery of LINEs to propagate. Retrotransposition contributes to individual genomic variations as well as distinctive features between lineages and species. The ability of retrotransposons to expand and colonize eukaryotic genomes has rendered them evolutionary successful and is responsible for creating genetic alterations leading to significant impacts on their hosts.

## 2. Regulation of Retrotransposon Activities

Novel integration and recombination events of active retrotransposons in the genome can cause spontaneous mutations and deletions, leading to dysregulation of neighboring genes and genome instability. Therefore, host cells have evolved a plethora of defense systems involving repressive epigenetic mechanisms to regulate these elements.

### 2.1. DNA Methylation

DNA methylation is one of the best-characterized epigenetic mechanisms. The addition of a methyl group on cytosine to generate 5-methylcytosine (5mC) functions to repress retrotransposons in various cell types [14,15,16]. Using mouse embryos deficient of DNA methyltransferase 1 (DNMT1), which catalyzes 5mC, Walsh et al. were the first to demonstrate that DNA methylation is necessary to silence intracisternal A particle (IAP) elements, a class II ERV [16]. More importantly, *DNMT1* mutants are embryonic lethal, potentially due to the derepression of genes and other TEs as a consequence of the global loss of 5mC [16,17,18,19,20]. These and other studies have established DNA methylation as the predominant pathway for retrotransposon silencing [16,21,22,23,24]. Interestingly, mammalian genomes undergo two waves of global DNA methylation reprogramming during embryogenesis: in preimplantation stages and gametogenesis [25]. At these developmental stages, the genome is de-methylated and then re-methylated, where transcriptional bursts of specific elements, such as L1s, human ERV subfamily K (HERV-K), and murine ERV subfamily L (MERV-L), coincide with dramatic 5mC loss [26,27,28,29]. These stage-specific reactivation events are essential to facilitate activation of the zygotic genome and the protection of the embryo. Importantly, most other retrotransposons remain silenced, indicating the presence of alternative repressive mechanisms.

### 2.2. Histone Modifications

Post-translational modifications (PTMs) of histone proteins play a crucial role in the regulation of retrotransposon activities. Numerous studies have identified several PTMs involved in the silencing of specific elements, including methylation of histone H3 at lysine 9 (H3K9), histone H3 at lysine 27 (H3K27), demethylation of H3 lysine 4 (H3K4), and deacetylation of H3K9 [30,31,32,33,34,35,36,37]. The sequence of retrotransposon subfamilies appears to, in part, determine its corresponding repression mechanism. For instance, in mouse embryonic stem cells (ESCs), H3K9 di-methylation (H3K9me2) deposited by G9a/GLP has a vital role in regulating L1 and class III ERVs, such as MERV-L [36,38,39], whereas H3K9 tri-methylation (H3K9me3), targets class I and II ERVs [33,34]. There are also other factors that direct specific epigenetic modifying enzymes to particular elements. For instance, double homeobox (Dux) and zinc finger protein 51 (Zfp51) regulate the establishment of stage specific H3K9me3 at LTR regions for silencing during early embryonic development [40]. Specifically, H3K9me3 is enriched at IAP, mouse type-D related retrovirus (MusD), and murine leukemia virus (MLV) elements and transcriptionally silences these sequences in a DNA methylation-independent manner [34]. This histone modification is catalyzed by the H3K9-specific methyltransferase SET Domain Bifurcated 1 (SETDB1), which is recruited by Krüppel-associated box-containing zinc-finger proteins (KRAB-ZFP) and KRAB-associated protein 1 (KAP1, also known as TRIM28). The KRAB-ZFP/KAP1 complex recognizes distinct sequences within retrotransposons to induce their repression. Interestingly, in order to defend against the rapidly evolving sequences of retrotransposons, KRAB-ZFP genes are also highly diverse. There are approximately 400 and 600 KRAB-ZFP genes in human and mouse genomes, respectively [41]. For instance, hominoid-specific KRAB-ZFP was recently identified to repress the primate-specific LTR12C elements in early embryonic and germline development [42]. Studies have also discovered that mouse lineage-specific KRAB-ZFP genes repress new families of retrotransposons in mice [43]. It has been suggested that an evolutionary arms race is ongoing between retrotransposons and their host organisms. As the host seeks to counteract the potentially harmful activities of retrotransposons by acquiring new silencing mechanisms, retrotransposons, in turn, continuously evolve in an effort to evade repression. Strikingly, approximately 15% of IAP elements evade H3K9me3-mediated repression due to their genetic divergence at the U3 regions of their LTRs. These proviruses can activate nearby genes through their putative enhancer functions, which has been co-opted to provide endogenous *cis*-regulatory elements during neural lineage establishment [44].

In addition to H3K9 methylation, H3K27 tri-methylation (H3K27me3) represents another crucial epigenetic modification involved in retrotransposon regulation. H3K27me3 is deposited by the Polycomb-repressive complex 2 (PRC2), which subsequently recruits Polycomb-repressive complex 1 (PRC1), facilitating chromatin compaction [45,46]. Notably, a study led by Walter and colleagues demonstrated that when mouse ESCs were cultured under specific conditions, they exhibited a significant loss of DNA methylation and H3K9me2, resembling the DNA methylation reprogramming that occurs during early embryogenesis. This loss of repressive marks was followed by an increase in H3K27me3 enrichment, which subsequently takes over to repress class III ERVs such as MERV-L elements [47]. Unlike H3K9me2, which has been shown to reciprocally regulate DNA methylation, this shows the necessity for precise and specific epigenetic mechanisms for retrotransposon silencing that operate independently of DNA methylation, particularly during reprogramming events. Similarly, during DNA demethylation in gametogenesis, H3K27me3 serves to suppress retrotransposons in the gonadal primordial germ cells [48].

**Figure 1:**
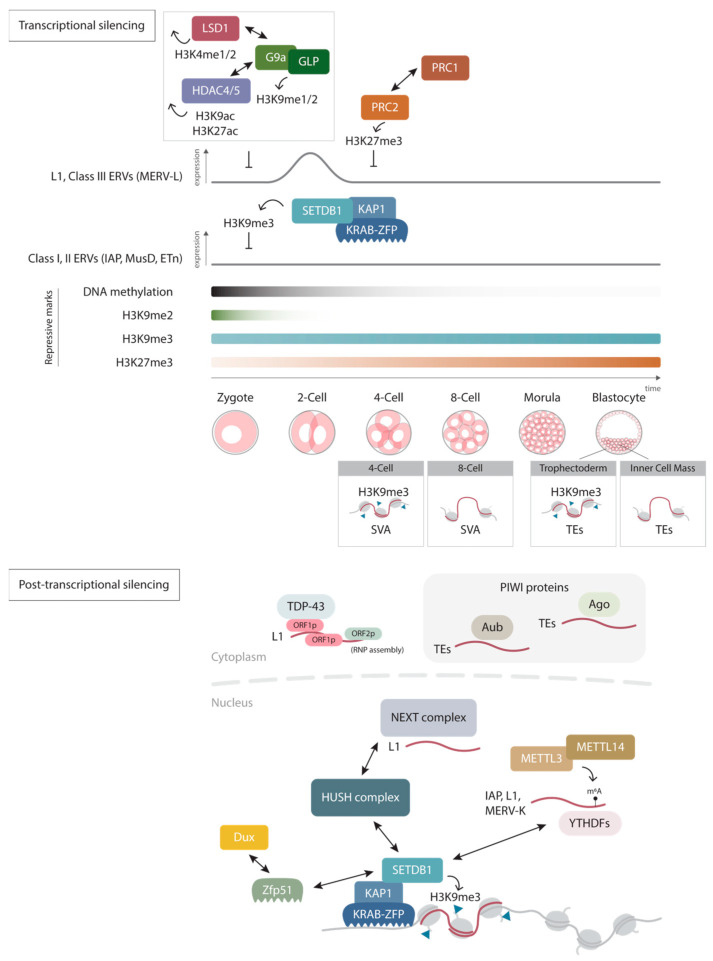
Regulation of retrotransposons. Retrotransposons can be regulated both transcriptionally and post-transcriptionally. In early mammalian development, different histone modifications such as H3K9me2, H3K9me3, and H3K27me3 supplement each other to repress retrotransposons during global DNA methylation reprogramming. Together with stage-specific cofactors, stage- and cell-type specific repression by H3K9me3 have also been described to facilitate precise spatial and temporal expression profiles for proper development. Host cells also employ different post-transcriptional silencing mechanisms in both the cytoplasm and inside the nucleus, including RNA decay via (P-Element-induced wimpy testis) PIWI proteins, TAR DNA-binding protein 43 (TDP-43), nuclear exosome targeting (NEXT) complex, and *N*6-methyladenosine (m6A) modifications. Some of which have also been implicated in regulating epigenetic modifications.

In addition to catalyzing repressive epigenetic marks, the removal of active modifications can also prevent retrotransposon activities. For example, lysine-specific demethylase 1 (LSD1, also known as KDM1A) represses retrotransposons not regulated by DNA methylation, through the demethylation of H3K4, a well characterized active mark [30]. In ESCs, the depletion of LSD1 results in extensive MERV-L reactivation through the increase in active histone marks such as H3K4 methylation and H3K27 acetylation (H3K27ac). It is noteworthy that the activation of these elements is associated with altered cell differentiation potentials. Similarly, histone deacetylases 4/5 (HDAC4/5), controlled by the PIM3 pathway, also targets MERV-L elements by removing H3K9 acetylation (H3K9ac) and H3K27ac [37]. While the mechanism has not been fully elucidated, both LSD1 and HDAC4/5 were demonstrated to facilitate H3K9 methylation by G9a in restricting TE activities [30,37]. Taken together, different histone modifications work both independently and in concert to precisely regulate retrotransposon activities (Figure 1).

### 2.3. Post-Transcriptional Regulatory Mechanisms

Besides epigenetic silencing, hosts have evolved multiple strategies for repressing TE transcripts. A crucial mechanism involves the action of P-Element-induced wimpy testis (PIWI)-interacting RNAs (piRNAs) and PIWI proteins, which play significant roles in both transcriptional and post-transcriptional silencing of retrotransposons with high specificity. First discovered in *Drosophila melanogaster*, piRNAs are small RNAs of approximately 24–30 nucleotides, which are loaded into Argonaute3 (Ago3) and Aubergine (Aub) proteins to target and degrade the complementary retrotransposon transcripts in the cytoplasm [49] (Figure 1). This pathway has been identified in humans and mice and functions exclusively in germ cells. piRNAs also transcriptionally repress retrotransposons within the nucleus by recruiting repressive epigenetic modifiers to specific elements [50,51].

Recent studies have described other nuclear RNA decay mechanisms that control TE transcripts. For instance, the epigenetic regulator human silencing hub (HUSH) complex, which assists in H3K9me3 maintenance by recruiting SETDB1, employs an RNA decay system that involves the nuclear exosome targeting (NEXT) complex to selectively degrade L1 transcripts in ESCs [52] (Figure 1). In addition, TAR DNA-binding protein 43 (TDP-43), a DNA/RNA-binding protein involved in RNA processing, inhibits L1 retrotransposition at the preimplantation stages of early embryogenesis [53] through the interaction between the N-terminal domain of TDP-43 and L1 ORF1 protein (ORF1p). The ability of TDP-43 to control TE transcripts is important in preserving genomic integrity, as its loss of function is associated with massive L1 genomic expansion, impaired embryonic growth, and lethality [54]. However, the mechanism by which TDP-43 neutralizes L1 retrotransposition remains unclear, with indications that it functions in a post-transcriptional manner.

The *N*6-methyladenosine (m6A) is the most prevalent modification in eukaryotic mRNA molecules that controls gene expression in diverse biological processes [55,56]. Methyltransferase like-3 (METTL3) and methyltransferase like-14 (METTL14) function as a complex to catalyze N6-adenosine methylation predominantly at stop codons and 3′ untranslated regions (3′ UTRs) in mammalian transcripts [57,58,59,60]. Multiple reader proteins have been reported to recognize m6A on messenger RNA (mRNA) and to determine their fate, including mRNA decay [61,62], mRNA stabilization [63], and enhanced translation [64]. For instance, YT521-B homology domain family (YTHDF) proteins can destabilize and degrade m6A-modified transcripts [65]. Transcriptome-wide sequencing aimed at mapping m6A-modified sites has demonstrated a reduced amount of m6A-enriched IAP, murine ERV subfamily K (MERV-K), and L1 transcripts in mouse ESCs, a phenomenon crucial for maintaining cell identity and dependent on YTHDF [66,67]. Therefore, retrotransposon transcripts can also be repressed by m6A RNA methylation (Figure 1). Intriguingly, YTHDF1 targets also mediate SETDB1-dependent H3K9me3 silencing of TEs in mouse ESC [67], thereby suggesting another layer of interplay between epigenetic transcriptional repression and RNA decay machinery in regulating retrotransposons.

## 3. Roles of Retrotransposons in Facilitating Higher-Order Nuclear Organization

**Figure 2:**
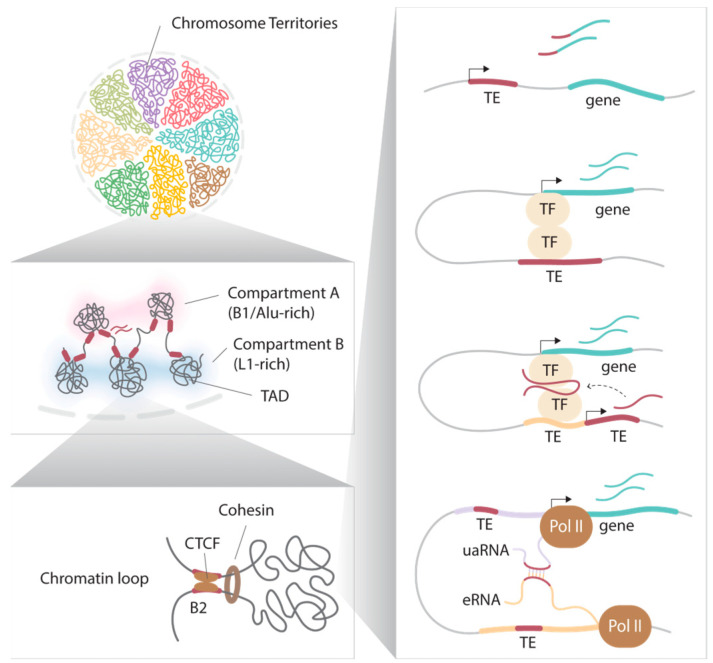
Retrotransposons in chromatin organization and gene regulation. Eukaryotic genomes are organized in a hieratical order, from chromosome territories to compartments and chromatin loops (**left**). A variety of retrotransposons shape the chromatin structure at different levels, such as the SINE B1/Alu and L1 transcription in compartments A and B, respectively; human ERV subfamily H (HERV-H) transcription at TAD boundaries; and SINE B2 elements enriched at TAD boundaries and chromatin loop anchors as binding sites for CCCTC-binding factor (CTCF) proteins to promote long-range chromatin interactions. Retrotransposons are also capable of altering transcriptions via distinct mechanisms (**right**). The elements can act as alternative promoters and enhancers. TE transcripts can facilitate TF binding to the target genes, and complementary enhancer RNA (eRNA) that is enriched with SINE Alu sequences can dictate enhancer–promoter pairing by forming duplex with upstream antisense promoter RNA (uaRNA).

Within the nucleus, the spatial organization and packaging of the genome exhibit a non-random nature, characterized by highly regulated hierarchical chromatin structures (Figure 2). During interphase, individual chromosomes adopt specific three-dimensional (3D) conformations and occupy distinct nuclear regions known as chromosome territories [68]. Chromatin regions can be further categorized into subnuclear compartments, termed A and B, which broadly represent euchromatic and heterochromatic domains. These compartments are defined through principal component analyses of high-throughput chromatin conformation capture (Hi-C) data, which assess spatial interactions between all genomic regions regardless of their linear distance. In general, loci in close spatial proximity tend to interact more frequently, and intra-compartment interactions are more common than inter-compartment interactions. Compartment A is typically associated with active loci located in the interior regions of the nucleus and exhibits higher levels of transcriptional activity and chromatin accessibility. In contrast, compartment B is more closely related to nuclear periphery localization, heterochromatic features, and compact chromatin structures [69,70]. Furthermore, it has been suggested that these subnuclear compartments may possess distinct biophysical properties. For instance, researchers have demonstrated phase separation, a phenomenon where dynamic spherical structures of chromatin bodies are formed [71,72,73]. These structures are believed to play a role in compartmentalization, keeping chromatin regions separate despite the absence of membranes. For example, the high local concentration of heterochromatin protein 1α (HP1α) promotes chromatin compaction by forming liquid droplets [72].

It is noteworthy that retrotransposons, such as LINEs and SINEs, also contribute to the organization of chromatin. Active transcription of L1, for instance, plays a role in regulating global chromatin accessibility during early mouse embryonic development [74]. In this context, L1 and B1/Alu transcriptions are critical for nuclear segregation [75]. Compartments rich in L1 elements are highly correlated with compartment B and are associated with the nuclear periphery and the nucleolus, known as lamina-associated domains (LADs) and nucleolus-associated domains (NADs), respectively. In contrast, euchromatic compartments rich in B1/Alu elements are associated with nuclear speckles. At a finer scale, computational analyses allow the definition of sub-compartments, including A1, A2, B1, B2, and B3, each exhibiting distinct epigenetic properties and transcriptional profiles across various cell types and developmental stages [70,76]. For example, evolutionarily expanded class II ERVs are notably enriched in the neuron-specific B2 sub-compartment in mice [77].

These compartments can be further segregated into kilobase-to-megabase sized topologically associated domains (TADs). Chromatin within the same TAD interacts more frequently than across TADs [78,79]. TADs play a role in transcriptional regulation by providing scaffolds for *cis*-regulatory interactions, where promoters and enhancers within the same TAD generally exhibit coordinated activities [78,79]. Furthermore, TADs are separated by distinct boundaries enriched with SINE Alu/B1 and B2 in mice, and SINE Alu elements in humans [78]. The disruption of specific TAD boundaries is associated with embryonic lethality and developmental defects [80,81]; therefore, maintenance of TAD structures is vital. Retrotransposons shape the chromatin landscape by acting as protein binding sites. CCCTC-binding factor (CTCF) is a critical component in the establishment of TAD boundaries and chromatin loops. CTCF functions as a transcriptional activator, a repressor, an enhancer blocker, or an insulator in a context-dependent manner [82]. In the regulation of higher-order chromatin structures, the loop extrusion model is a widely accepted mechanism where CTCF binding at specific sites, together with the cohesin complex, can form the anchors of a chromatin loop [83,84]. One-third of murine SINE B2 [85,86], as well as a large portion of B2-related ancient SINE B3 and B4 elements [86], carry CTCF-binding sequence motifs. These SINE B2 and B2-related genomic sequences serve as CTCF docking sites, regulated by DNA methylation, and control chromatin interactions to shape the 3D nuclear architecture [87,88]. Additionally, retrotransposon transcription is also involved. Although ERVs are usually not enriched at the TAD boundary, a subset of human ERV subfamily H (HERV-H) is transcriptionally active in human pluripotent stem cells (hPSCs) to create cell-type and species-specific TAD boundaries [89]. Moreover, the insertion of HERV-H is sufficient to introduce de novo TAD boundaries in other genomic locations [89]. Notably, the same TAD boundaries have been detected in other closely related species, which also possess HERV-H integrations. Although the mechanism remains unclear, it has been proposed that high levels of transcription by RNA polymerase II impact cohesin complex positioning to form TAD boundaries. Similarly, mammalian-wide interspersed repeats (MIRs), which are tRNA-derived SINEs, also participate in tissue-specific domain boundary establishment, likely through recruitment of RNA polymerase III [90].

Given their capacity for mobilization, retrotransposons have produced TE variants (TEVs) that differ in integration sites between species or are polymorphic within a species. The identification and characterization of TEVs can provide a deeper understanding of the roles of these elements in the host cells. In the context of genome architecture, retrotransposition of specific elements contributes to the expansion of the repertoire of potential CTCF binding sites in the host genomes. Throughout evolution, ancient CTCF binding sites derived from retrotransposon TEVs can underlie species divergent higher-order chromatin structures [86]. Notably, using deep whole genome sequencing data, Nellåker et al. defined CTCF-associated TEVs from all TEVs (*n* = 103,798) between 18 mouse strains [91]. A subset of these sites was associated with differential transcriptional levels. For example, an IAP-I provirus, which was defined in 15 of the mouse strains, harbored a CTCF binding site. The presence of this element is associated with higher expression of the nearby *Slc36a1* gene, presumably by establishing differential higher-order chromatin interactions [91]. In addition, a recent report utilized long-read sequencing to analyze the genomes of 20 inbred mice of distinct genetic backgrounds. The number of TEVs they discovered (*n* = 99,349) [92] is almost the same as the totality of previously reported TEVs [91], highlighting the problems encountered by short-read sequencing. The integration of these TEVs was polymorphic between animals of the same species. A proportion of these polymorphic retrotransposons, including L1 and IAP elements, are correlated with differential levels of chromatin accessibility [92]. In particular, a de novo insertion of an early transposon (ETn) class II ERV in the CAST/EiJ mouse strain is coupled with strain-specific higher chromatin accessibility and increased expression of the adjacent *SLc47a2* gene as compared with nine other strains lacking the provirus [92]. Taken together, a substantial body of evidence supports the notion that retrotransposons contribute to genome diversity and play a role in shaping the dynamic 3D architecture of the genome.

## 4. Roles of Retrotransposons in Development

Although most retrotransposons have lost their capacity to mobilize or are silenced by epigenetic mechanisms, a proportion still harbors *cis*-regulatory modules and splicing donor/acceptor sites that increase host transcriptome variability. Retrotransposons have been evolutionarily co-opted to function as alternative promoters for crucial host gene networks to drive the lineage-specific expression [33,39,93,94] (Figure 2). In mouse early embryonic development, MERV-L-LTR elements serve as promoters for specific two-cell (2C) stage genes during zygotic genome activation (ZGA) and generates chimeric transcripts that specify primitive endoderm and trophectoderm lineages [28,39]. Other elements function similarly at different developmental timepoints. For example, a mouse-specific MT2B2-LTR drives a transient isoform of the *Cdk2ao1* gene (Cdk2ap1^ΔN^) in preimplantation embryos to promote proliferation and implantation [95]. Since retrotransposons possess different transcription factor (TF) binding sites, they can also serve as enhancers in a context-dependent manner [96] (Figure 2). In early human development, certain retrotransposons undertake stage- and lineage-specific enhancer roles and exhibit dynamic H3K9me3 enrichment, which selectively represses them when their activity is not required [97]. In particular, SINE-VNTR-Alu (SVA) elements promote human ZGA by facilitating the interaction between SVA-derived enhancers and ZGA gene promoters in eight-cell (8C) embryos. Notably, these same elements are repressed in four-cell (4C) embryos [97]. On the other hand, in the inner cell mass (ICM), some hominoid-specific retrotransposon-derived regulatory elements provide TF binding sites for pluripotent genes and are enriched with de novo H3K9me3 in the trophectoderm [97] (Figure 1). Overall, these studies demonstrated the domestication of retrotransposons and how they are epigenetically regulated during early development.

In addition to their *cis*-regulatory roles, retrotransposon transcripts play pivotal roles in directing host cell fates during preimplantation development [98]. As mouse embryogenesis progresses from the totipotent to pluripotent states, a surge in MERV-L transcripts suppresses 2C gene expression, albeit through a mechanism that remains unclear. Furthermore, L1 RNA, which is also abundantly present in early murine development, interacts with Nucleolin and KAP1 to exit the two-cell stage by repressing Dux, ultimately promoting self-renewal and developmental potency [99]. On the contrary, HERV-H exhibits high expression levels in human pluripotent stem cells, actively promoting pluripotency while repressing differentiation genes [100]. Mechanistically, HERV-H transcripts have the capacity to induce the expression of neighboring genes and long noncoding RNAs (lncRNAs) by interacting with coactivators and pluripotency factors like P300 and octamer-binding transcription factor 4 (OCT4) [100] (Figure 2). Additionally, the elevated expression of HERV-H contributes to the repression of harmful young retrotransposons, possibly by promoting the expression of the *apolipoprotein B mRNA editing enzyme and catalytic polypeptide-like 3* (*APOBEC3*) genes [101].

Intriguingly, retrotransposons with detrimental effects on host cells also play a role in early development. Utilizing single-cell RNA sequencing (scRNA-seq), Singh et al. proposed the concept of “REject cells” in human embryos, a previously undefined cell population enriched with active young retrotransposons, DNA damage, and apoptotic signatures. These cells are selectively eliminated from the developmental process and contribute to maintaining the overall fitness of the embryos [101]. In mice, however, the authors did not detect the presence of any REject cells. Further investigations are warranted given the differences in heterogeneity between human and mouse embryos.

## 5. Involvement of Retrotransposons in Diseases

### 5.1. Placental Development and Disease

In placental mammals, retrotransposons are crucial for establishing important gene expression patterns in the placenta, including the *SYNCYTIN* gene, which is derived from the *env* gene of human ERV subfamily W (HERV-W) elements [102,103]. In addition, ERVs are involved in regulating the expression of the *pregnancy-specific glycoprotein* (*PSG)* genes in syncytiotrophoblasts, which are required to facilitate the remodeling of the maternal–fetal interface for proper placentation [104,105,106]. Specifically, LTR8B elements serve as enhancers for *PSGs* in normal pregnancy, and knockout of the LTR8B element in the intron of *PSG5* downregulates the gene’s expression [107]. Interestingly, upon infection with Severe Acute Respiratory Syndrome Coronavirus 2 (SARS-CoV-2), these retrotransposon-derived enhancers are dysregulated and affect their target genes. For instance, in the placental cells from patients infected by SARS-CoV-2 during pregnancy, the LTR8B elements show marked reduction in active epigenetic signatures and decreased expression of 7 out of the 10 *PSGs* [106]. The changes in retrotransposon-derived enhancer activities may also play a role in endothelial dysfunction of COVID-19 patients via *vascular endothelial growth factor A* (*VEGFA*) and *endoglin* (*ENG*) gene dysregulation. These genes are known to be involved in pregnancy complications such as preeclampsia. Moreover, retrotransposons can impact innate immunity, which is important for fetal protection in hemochorial placentas. Upon viral infection, innate immunity is an essential line of defense against invading pathogens. This mechanism can be initiated by recognizing dsDNA or double-stranded RNA (dsRNA) in the cytoplasm, which in turn triggers signaling pathways for interferon (IFN) production [108]. While type I and II IFNs pose high risks of pregnancy complications [109], type III IFNs protect the fetus via an unclear mechanism. A recent study discovered that the expression of SINE elements, including C19MC Alu dsRNA in humans and C2MC B1 dsRNA in mice, drives type III IFN expression and antiviral protection in placenta cells [110]. Wickramage and colleagues demonstrated that these lineage-specific SINEs resulted in convergent evolution of hemochorial placental antiviral immunity to ensure species survival.

### 5.2. Neurodegenerative Diseases

Retrotransposons contribute to brain development and evolution through its *cis*-regulatory function. For example, DNA demethylation activates L1s to drive neural gene expression as alternative promoters in human neural progenitor cells (NPCs) [111]. Moreover, human-specific L1-derived lncRNA *LINC01876* expression is crucial for proper neurodifferentiation and cerebral organoid development [112]. Interestingly, species-specific domestication of MER41 HERVs, which are enriched in the promoter of intellectual disability-associated genes, has been proposed to account for the cognitive differences between humans and chimpanzees [113]. Notably, somatic mobilizations of TEs have been observed in numerous neurodegenerative disorders. These include instances of derepression and/or retrotransposition of L1 elements associated with Parkinson’s disease [114], schizophrenia [115], Alzheimer’s disease (AD) [116], both L1 and HERV-K with amyotrophic lateral sclerosis (ALS) [117,118,119], and HERV-W with multiple sclerosis (MS) [120]. Additionally, in the *Drosophila* AD model, the dysregulation of Gypsy, Copia, and Het-A retrotransposons has been identified [116].

In humans, the loss or mutation of TDP-43 has been observed in the brains of patients with ALS, AD, and Parkinson’s disease [121,122,123,124,125,126]. A study conducted in mouse embryonic stem cells by Li et al. suggests a connection between neurodegenerative pathology and the accumulation of L1 elements resulting from TDP-43 mutation. This highlights L1 retrotransposition-related elements, including TDP-43 and L1 reverse transcriptase, as potential therapeutic targets for such complex disorders.

TE transcription may also play a role in the pathogenesis of Huntington’s disease (HD) [127]. In a *Drosophila* HD model, inhibiting reverse transcription activity has been shown to rescue HD-related neurodegenerative phenotypes in the eyes and to extend the lifespan of HD flies, potentially by restoring genome stability. While animal models have provided valuable insights into human developmental defects and diseases, they are limited by genome composition and species-specific TEs. Moreover, complex phenotypes involving diverse gene regulation can also be attributed to TE polymorphism. Recently, Modenini et al. investigated human polymorphic non-reference TEs (nrTEs) and identified evolutionarily young nrTE candidates that may potentially increase the risk of schizophrenia [128]. However, the underlying mechanisms remain elusive without functional validations. A deeper understanding of how these polymorphic nrTEs impact cognitive diseases is crucial for advancing complex disease prevention and treatment strategies.

### 5.3. Cancer and Potential Cancer Therapies

The epigenomes of cancer cells are radically reprogrammed to favor tumor survival and progression. For instance, global DNA hypomethylation and focal promoter hypermethylation have long been observed in various cancers [129,130,131]. Particularly, derepressed somatic retrotransposons which escaped DNA methylation, such as ERVs, L1, and Alu elements, are commonly found in many human cancers [132,133,134,135], and contribute to genome instability, inactivation of tumor suppressor genes, and activation of oncogenes through their *cis*-regulatory roles [134,136,137]. In recent studies employing scRNA-seq and proteogenomic methodologies, it has been identified that the human leukocyte antigen is encoded by L1, LTR, and SVA elements, particularly within glioblastoma [138]. The derepression of TEs in tumors is generally a consequence of DNA hypomethylation. The limited expression of these TE-derived peptides in healthy samples suggests that TEs could also serve as potential targets for cancer immunotherapy. Intriguingly, the reactivation of retrotransposons due to DNA hypomethylation has been recently reported to suppress tumor progression and is associated with a better prognosis. The widespread derepression of HERV-H in tumors leads to the increased expression of multiple KRAB-ZFP genes, which in turn silences genes related to proliferation [139]. Manipulating the epigenome has previously been proposed as one of the anti-tumor mechanisms through viral mimicry induction. Specifically, the inhibition of DNMTs in melanoma and colorectal cancer activates retrotransposons, reducing cell proliferation. The reverse-transcribed dsRNAs subsequently elevate the expression of interferon-responsive genes, triggering an anti-viral response [140,141]. Moreover, retrotransposons are also known to regulate transcription through their RNAs. An interaction between complementary enhancer RNA (eRNA) and upstream antisense promoter RNA (uaRNA) can potentially form duplexes and promote enhancer–promoter looping (Figure 2). Utilizing RNA in situ conformation sequencing (RIC-seq), retrotransposons, particularly Alu elements, have been found to be enriched in enhancer–promoter–RNA interactions (EPRIs) that dictate proper enhancer–promoter pairing [142]. Liang et al. demonstrated that Alu variants contribute to tumorigenesis, at least in part, by impairing EPRIs. This sheds light on additional potential avenues for retrotransposon-related cancer therapeutic strategies in the future.

## 6. Conclusions and Perspectives

With the advancement of functional genomic technologies such as scRNA-seq, RIC-seq, and Hi-C, our understanding of how retrotransposons participate in host genomes’ functions and their evolutionary expansion has significantly improved. Precise spatial and temporal retrotransposon activities, mediated by various epigenetic mechanisms, are crucial for essential physiological processes. Disruptions in these regulatory mechanisms, both at the transcriptional and post-transcriptional levels, caused by intrinsic and extrinsic factors like viral infections, can contribute to disease pathogenesis. Therefore, modulating retrotransposon activities and comprehending their functions offer new perspectives on diagnostic tools and therapeutic opportunities.

However, findings related to TEVs have limitations in translational research due to the influence of polymorphic ERVs on intra-species divergence [143]. To address this challenge, the innovation of sequencing technologies, such as long-read sequencing, has identified nearly twice as many new TEVs, some of which are associated with phenotypic differences. Investigating these elements was previously challenging due to the constraints of short-read sequencing. Recent resources, including the Telomere-to-Telomere (T2T) consortium and the Human Pangenome Reference Consortium (HPRC), have emerged to create complete assemblies and diversified human reference genomes. These references facilitate the exploration of variations in unknown genomic sequences, such as pericentromeric and centromeric DNA, as well as retrotransposons. Therefore, future research using long-read sequencing data will provide opportunities to further our understanding in the regulation of retrotransposons and their roles during development and disease.

## Data Availability

No new data were created or analyzed in this article. Data sharing is not applicable.
